# Extranodal Organ Involvement in Chronic Lymphocytic Leukemia: Clinical Features and Outcomes With Bruton Tyrosine Kinase and BCL‐2 Inhibitors

**DOI:** 10.1002/cam4.72341

**Published:** 2026-10-04

**Authors:** Finn J. Schulze, Evgenii Shumilov, Akhil Behringer, Andrea Kerkhoff, Matthias Eckhardt, Simone Bätker, Markus Maulhardt, Georg Lenz, Alexander Pohlmann

**Affiliations:** ^1^ Department of Medicine A, Hematology, Oncology and Pneumology University Hospital Münster Münster Germany; ^2^ Oncology Practice and Day Clinic Gelsenkirchen Germany; ^3^ MVZ Media Vita Münster Hematology and Oncology Practice Münster Germany; ^4^ Department of Hematology and Medical Oncology University Hospital Göttingen Göttingen Germany

**Keywords:** Bruton tyrosine kinase inhibitor, chronic lymphocytic leukemia, extranodal involvement, venetoclax

## Abstract

**Background:**

Although chronic lymphocytic leukemia (CLL) typically follows an indolent course involving blood, lymph nodes, and bone marrow, extranodal organ involvement is rare and poorly characterized.

**Methods:**

We retrospectively analyzed 19 patients with histologically confirmed extranodal CLL or small lymphocytic lymphoma (SLL), assessing organ involvement, clinical features, genetic risk factors, and response to therapy.

**Results:**

Different organs were affected, most frequently the kidneys (7/19). Median age at initial CLL diagnosis was 54 years, median time from CLL diagnosis to extranodal manifestation was 3 years. Genetic risk factors were comparable to typical CLL cohorts. Per treatment episode, exploratory overall response rate (ORR) for Bruton tyrosine kinase inhibitors (BTKi) was 88.9% (8/9); median progression‐free survival (PFS) from treatment initiation was 29 months. For venetoclax‐based regimens, ORR was 83.3% (5/6) and median PFS 52 months.

**Conclusions:**

These data suggest meaningful activity of BTK and BCL‐2 inhibitors in CLL/SLL with extranodal organ involvement.

## 1 Introduction

Chronic lymphocytic leukemia (CLL) typically follows an indolent course with involvement of lymph nodes, blood, and bone marrow. In contrast, extranodal organ involvement is rare, but it can cause substantial organ‐specific morbidity [1]. Contemporary data on the outcomes of these patients remain limited and are largely restricted to case reports or small heterogeneous series, often without histologic confirmation or detailed information on modern targeted treatment exposure [1, 2].

Here, we report clinical features and outcomes of novel CLL therapies in a cohort of patients with histologically confirmed extranodal CLL/small lymphocytic lymphoma (SLL).

## 2 Methods

This retrospective study included 19 patients with CLL/SLL and histologically confirmed extranodal organ involvement who were documented at the University Hospital of Münster between May 2000 and January 2026. Patients were eligible if institutional documentation was available in the context of diagnostic evaluation, treatment, follow‐up, or specialist consultation at our center. Clinical data were extracted from institutional records and available external medical reports. In accordance with the current WHO classification, CLL and SLL are considered a single disease entity and are collectively referred to as CLL throughout this manuscript unless otherwise specified. Clinical data were obtained through review of electronic medical records and supplementary patient‐related documentation. No additional samples were taken and no changes were made to the patients' treatment. The project was conducted in accordance with the Declaration of Helsinki and was approved by the Ethics Committee of Westphalia Lippe (2025‐828‐f‐S); the requirement for informed consent was waived due to the retrospective design.

Progression‐free survival (PFS) was estimated by the Kaplan–Meier method using IBM SPSS Statistics (version 31.0; IBM). PFS was defined as time from initiation of the respective treatment episode to progression or death from any cause; patients without an event were censored at last follow‐up. Overall response rate (ORR) was calculated as the proportion of treatment episodes achieving complete or partial response according to iwCLL 2018 criteria [3]. In addition, response of extranodal organ involvement was assessed based on organ‐specific clinical, radiologic, and/or pathologic follow‐up documentation, as applicable. Given the retrospective design and heterogeneity of affected organs, no centralized organ‐specific response assessment was performed. Analyses were performed per treatment episode. For Bruton tyrosine kinase inhibitor (BTKi) monotherapy, all available agents were pooled (ibrutinib, acalabrutinib, zanubrutinib, and pirtobrutinib). Two additional patients received ibrutinib prior to the development of extranodal disease; these episodes were excluded from class‐specific efficacy analyses as the BTKi was not initiated to treat extranodal involvement. Venetoclax‐based regimens were pooled for ORR and PFS analyses. One venetoclax‐based episode was excluded from ORR analyses due to missing post‐treatment assessment, and one concomitant acalabrutinib‐plus‐venetoclax episode was excluded from class‐specific ORR and PFS analyses to avoid attribution to a single drug class. All efficacy analyses were descriptive and exploratory, and no formal comparison between BTKi‐ and venetoclax‐based regimens was intended given the small and heterogeneous sample. Median follow‐up was estimated using the reverse Kaplan–Meier method.

## 3 Results

### 3.1 Patient Characteristics

Patient characteristics are presented in Table 1. Figure 1 graphically shows the course of the disease under treatment for distinct patients.

**TABLE 1:** Patient‐level characteristics of 19 patients with histologically confirmed extranodal CLL/SLL.

Sex	Age at CLL dx (in years)	Age at FOM (in years)	Timing of FOM	Organ(s)	Selected genetic findings	BTKi and/or venetoclax‐based therapy
M	42	42	At diagnosis	Kidney	Progression: IGHV mutated, *TP53* unmutated, 46, XY, del(11q22.3), del(13q14.2), *BIRC3* wildtype	2nd line: **Acalabrutinib**
F	36	39	After watch and wait	Kidney	*TP53* unmutated, no del(17p), trisomy 12	1st line: **Obi/Ven**
M	68	72	After watch and wait	Kidney	IGHV mutated, *TP53* unmutated, del(13q14)	2nd line: **Obi/Ven**
F	34	38	After watch and wait	Kidney	IGHV mutated, *TP53* unmutated, trisomy 12, del(13q14.1)	Only non‐BTKi/BCL‐2 inhibitor therapy
F	68	72	After watch and wait	Kidney	IGHV mutated, del(17p) (*TP53*), monosomy X, del(6q21), monosomy 6, trisomy 12cen/q15, del(13q14.2)	1st line: **Zanubrutinib**
M	72	80	After 2nd therapy line	Kidney	No del(17p)	2nd line: **Ibrutinib** (before extranodal manifestation)
F	30	42	After 2nd therapy line	Kidney, CNS	Initially: IGHV unmutated, *TP53* unmutated, 46, XX Progression: *TP53* mutated, *SF3B1* mutated, del(13q) Subsequently: complex karyotype	3rd line: **Ibrutinib** 4th line: **Obi/Ven** 5th line: **Pirtobrutinib**
M	48	48	At diagnosis	CNS	Normal karyotype	Only non‐BTKi/BCL‐2 inhibitor therapy
M	46	46	At diagnosis	Pleura, CNS	Progression: IGHV unmutated, *TP53* unmutated, del(11q22.3), trisomy 12	2nd line: **Ibrutinib** 3rd line: **R‐Ven** 4th line: **Pirtobrutinib**
F	66	66	At diagnosis	Breast	IGHV mutated, *TP53* unmutated, Karyotype 45,X,‐X	Only non‐BTKi/BCL‐2 inhibitor therapy
F	52	52	At diagnosis	Breast	n.a.	No systemic therapy (watch and wait)
M	54	62	After watch and wait	Paranasal sinus	*TP53* unmutated, *SF3B1* gene mutation, *ATM* gene mutation, BTK gene mutation (p.C481S and p.C481R substitution)	2nd line: **Ibrutinib** 3rd line: **R‐Ven**
F	64	64	At diagnosis	Nasal skin	*TP53* unmutated, no del(17p), del(11q22), monosomy 6	Only non‐BTKi/BCL‐2 inhibitor therapy
M	76	76	At diagnosis	Orbit	Progression: IGHV mutated, *TP53* unmutated, 46,XY	1st line: **Obi/Ven**
M	43	51	After 5th therapy line	Liver	Progression: *TP53* unmutated, del(11q) After Richter‐transformation: complex karyotype	4th line: **Ibrutinib** (before extranodal manifestation) 6th line: **Venetoclax** **Richter transformation**
F	72	72	At diagnosis	Gastric	IGHV mutated, *TP53* unmutated, no complex karyotype	1st line: **Acalabrutinib** , starting in the 3rd cycle: **Acalabrutinib + Venetoclax**
F	49	66	After 3rd therapy line	Labia	n.a.	4th line: **Zanubrutinib**
M	69	82	After 1st therapy line	Gallbladder	IGHV unmutated, *TP53* mutated, 47,XY,+12	Only non‐BTKi/BCL‐2 inhibitor therapy
F	59	59	At diagnosis	Hypopharynx	IGHV mutated, *TP53* unmutated, *SF3B1* deletion	No systemic therapy (watch and wait)

*Note:* The column on the right shows whether patients received BTKi and/or venetoclax‐based therapies. Additionally, the extranodal manifestation is classified chronologically in relation to the therapies.

Abbreviations: BTKi, Bruton tyrosine kinase inhibitor; dx, diagnosis; FOM, first organ manifestation; Obi/Ven, obinutuzumab and venetoclax; Pt., patient; R‐Ven, rituximab and venetoclax.

**FIGURE 1:**
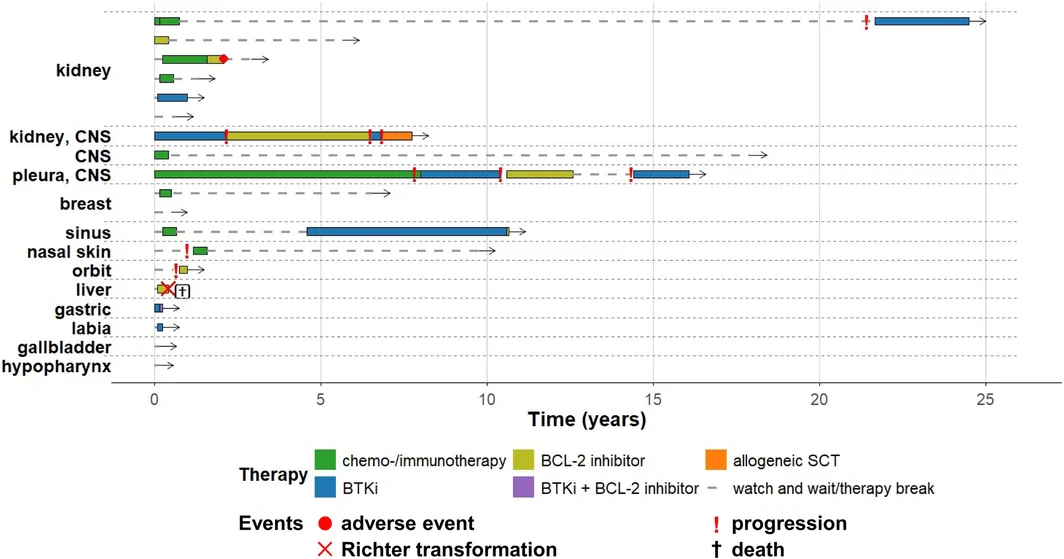
Clinical course of each patient after extranodal manifestation. Swimmer plot of individual clinical courses after first biopsy‐proven extranodal organ involvement (time 0). BTKi therapies were pooled, including ibrutinib, acalabrutinib, zanubrutinib, and pirtobrutinib. Similarly, the BCL‐2 inhibitor‐based therapies include obinutuzumab/venetoclax and rituximab–venetoclax. Chemo‐ and/or immunotherapies include FCR, BR, and others. The arrows indicate that the last therapy displayed is currently still being continued. The order of the patients corresponds to the order in Table 1.

Median age at CLL/SLL diagnosis was 54 years (range 30–76). Ten patients were female.

Nine of 19 patients presented with extranodal organ involvement at initial diagnosis. Among the remaining patients, extranodal manifestations developed after a median of 8 years (IQR 4‐11; maximum 17 years) following initial diagnosis. Five of these 10 patients presented extranodal manifestations after initial watch and wait strategy, 3/10 after one or two therapy lines, and 2/10 after three or more therapy lines.

In our cohort, *TP53* status was available for 16/19 patients, and 3/16 had del(17p) and/or a *TP53* mutation. Immunoglobulin heavy‐chain variable region (IGHV) mutational status was available in 11/19 patients and was mutated in 8/11. Del(11q) or a mutation in the *ATM* gene was detected in 5/19 patients. Karyotype information was available in 8/19 patients; 2/8 had a complex karyotype.

Genetic findings were reported irrespective of whether they were detected at initial diagnosis or later during the disease course. However, clonal evolution was observed in our cohort. In one illustrative case, *TP53* status and karyotype evolved from wild‐type/normal to a *TP53* mutation with complex karyotype in parallel with disease progression after multiple lines of therapy.

The most frequent manifestation was renal infiltration (7/19), followed by meningeal lymphomatosis (3/19). In the given patient cohort, apart from kidney and CNS, a wide range of additional organs were affected, including skin, breast, labia, liver, gallbladder, stomach, pleura, orbit, hypopharynx, and paranasal sinuses.

### 3.2 Treatment and Outcomes

In our cohort, seven patients received venetoclax‐based regimens, predominantly combined with anti‐CD20 antibodies. BTKi therapy included ibrutinib (*n* = 3) and the second‐generation agents acalabrutinib (*n* = 2) and zanubrutinib (*n* = 2); two patients received the non‐covalent BTKi pirtobrutinib. Four patients were treated sequentially with a BTKi followed by venetoclax. One patient received concomitant BTKi and BCL‐2 inhibition, with ongoing treatment and a response observed at last follow‐up.

For class‐level analyses, BTKi agents (ibrutinib, acalabrutinib, zanubrutinib, and pirtobrutinib) were pooled. Given the small sample size, all efficacy analyses are exploratory. Per treatment episode (*n* = 9), median PFS was 29 months (95% CI 22.8–35.2). Partial or complete response was observed in eight out of nine treatment episodes.

BTK resistance mutations were detected in one patient with extranodal involvement of the paranasal sinuses, the only patient for whom BTK resistance testing was available. The mutations resulted in p.C481S and p.C481R substitutions, which are associated with resistance to covalent BTK inhibitors and thus prompted a change in treatment to a venetoclax‐based regimen.

Venetoclax‐based regimens were pooled accordingly. Per treatment episode (*n* = 7), median PFS was 52 months (95% CI 12.2–91.8). Among evaluable episodes, five out of six demonstrated either a partial or complete response.

Data on venetoclax‐associated resistance mutations were unavailable.

Patients with renal infiltration showed heterogeneous clinical courses, ranging from prolonged disease control with a single therapy to repeated relapses requiring multiple treatment lines.

Two out of three patients with meningeal lymphomatosis in our cohort suffered multiple relapses and required four or more lines of therapy.

One patient developed Richter transformation after multiple lines of therapy, including prior BTKi exposure.

The median follow‐up for BTKi therapy was 34 months (*n* = 9). For venetoclax‐based regimens, median follow‐up was 68 months (*n* = 6). At last follow‐up, 18 patients were alive. One patient died from sepsis following allogeneic stem cell transplantation for Richter transformation.

## 4 Discussion

In our cohort, median age at CLL/SLL diagnosis was 54 years, compared with 70 years reported in the literature [4]. Whether this reflects a biological predisposition or a biopsy bias, with clinicians being more inclined to obtain histological confirmation in younger patients, cannot be determined from our data.

Although some patients presented extranodal manifestation at initial diagnosis, others developed extranodal disease after up to 17 years. This is consistent with prior reports describing extranodal disease as both an early and late manifestation [1].

In terms of genetic risk factors, the frequency of *TP53* aberrations (3/16) was within the range reported for unselected CLL populations, suggesting no marked enrichment in patients with extranodal organ involvement [5]. Similarly, the frequency of del(11q) or a mutation in the *ATM* gene was within the ~10%–25% range reported for unselected CLL cohorts [6]. In contrast, the proportion of IGHV‐mutated cases (8/11) was numerically higher than typically reported in unselected CLL cohorts (~50%–60%) [7]. Given the known association of IGHV‐mutated status with younger age at diagnosis [7], this likely reflects the substantially younger age in our cohort rather than a biological enrichment specific to extranodal involvement. Karyotype data were available for eight patients, two of whom had a complex karyotype, consistent with published rates of ~14%–34% [8].

In the given cohort, the kidney was the most common extranodal manifestation, followed by meningeal lymphomatosis. This differs from previous reports, where skin and CNS involvement are the most reported extranodal manifestations [1, 2]. The relatively high proportion of renal involvement may partly reflect the diagnostic approach in specialized centers, where kidney biopsy is readily performed in patients with otherwise unexplained changes in renal function. Therefore, our findings do not necessarily indicate that the kidney is more commonly affected by CLL. The genetic findings among these patients were heterogeneous, with no clear pattern suggesting a specific genetic risk for renal involvement.

Although chemoimmunotherapy has classically been used in CLL for more than two decades, BTKi and BCL‐2 inhibitors were introduced more recently [9, 10, 11, 12]. Hence, there is only limited data on the efficacy of these novel therapies in patients with extranodal organ involvement in CLL/SLL.

In our cohort, median PFS with BTKi therapy (29 months) was numerically shorter than reported in pivotal trials of covalent BTKi in relapsed/refractory CLL (44 months in RESONATE [9]). This likely reflects the heterogeneity of treatment episodes, which spanned first‐ through fifth‐line therapy and included non‐covalent BTKi administered after prior covalent BTKi exposure—a setting in which substantially shorter PFS has been documented [13].

Median PFS with venetoclax‐based regimens (52 months) was consistent with outcomes reported in unselected CLL cohorts [14, 15].

Taken together, response rates and PFS in our cohort argue against a specific pattern of treatment resistance in extranodal CLL/SLL.

Given the small and heterogeneous cohort and the non‐randomized, treatment episode–based nature of the analysis, the observed response rates and PFS estimates should not be interpreted as evidence of differential efficacy between BTKi‐ and venetoclax‐based regimens.

Prior studies have suggested that survival is dependent on the type of organ infiltration, with CNS involvement being associated with an unfavorable prognosis [2]. In line with this observation, two out of three patients with meningeal lymphomatosis in our cohort experienced recurrent disease progression.

Ratterman et al. [2] suggested that patients who develop extranodal organ involvement at relapse may have a poorer prognosis than those presenting with extranodal disease at initial diagnosis. In our cohort, this pattern was not observed. However, available data on extranodal involvement in CLL are limited, and previous reports largely predate the widespread use of BTKi and venetoclax.

Limitations include the retrospective single‐center design, the inherently small sample size given the rarity of extranodal CLL, heterogeneous genetic data availability, and the per‐episode analysis in which multiple non‐independent treatment episodes from the same patient may introduce bias.

## 5 Conclusions

Extranodal organ involvement in CLL/SLL spans a broad anatomical spectrum, with renal infiltration being the most frequent manifestation in this histologically confirmed cohort. Genetic risk features were not clearly enriched compared with conventional CLL. In exploratory treatment‐episode‐level analyses, BTK inhibitor‐ and BCL‐2 inhibitor‐based regimens showed meaningful activity without an obvious signal of extranodal‐specific treatment resistance.

## Author Contributions

**Finn J. Schulze:** writing – original draft, investigation, conceptualization, methodology, visualization, writing – review and editing, formal analysis, software, data curation, project administration, resources, validation. **Evgenii Shumilov:** writing – review and editing, resources, supervision. **Akhil Behringer:** writing – review and editing, resources. **Andrea Kerkhoff:** writing – review and editing, resources. **Matthias Eckhardt:** writing – review and editing, resources. **Simone Bätker:** writing – review and editing, resources. **Markus Maulhardt:** writing – review and editing, resources. **Georg Lenz:** writing – review and editing, supervision, resources. **Alexander Pohlmann:** conceptualization, writing – review and editing, supervision, resources, project administration, methodology, data curation, investigation, validation.

## Funding

No external funding was received for this study. The research was conducted using the authors' institutional resources.

## Ethics Statement

The project was approved by the Ethics Committee of Westphalia Lippe (2025‐828‐f‐S).

## Conflicts of Interest

Finn J. Schulze received congress support from Lilly. Akhil Behringer received travel support from Sanofi and Lilly. Markus Maulhardt received travel and congress support from AbbVie, Kite Gilead, Sobi, and Incyte and honoraria from Kite Gilead and Lilly. Georg Lenz received honoraria from AbbVie, ADC, AstraZeneca, BeOne, Bristol Myers Squibb, Eli Lilly, Exscientia, Flindr, Genmab, Gilead, GSK, Hexal/Sandoz, Incyte Corporation, Miltenyi, MSD, Novartis, PentixaPharm, Pierre Fabre, Roche, and Sobi; received consultancy fees and/or participated in advisory boards for AbbVie, ADC, AstraZeneca, BeOne, Bristol Myers Squibb, Eli Lilly, Exscientia, Flindr, Genmab, Gilead, GSK, Hexal/Sandoz, Incyte Corporation, Miltenyi, MSD, Novartis, PentixaPharm, Pierre Fabre, Roche, and Sobi; received research support from AbbVie, AstraZeneca, Gilead, Novartis, and Sobi; and received support for travel, accommodation, and/or expenses from AbbVie, AstraZeneca, BeOne, Bristol Myers Squibb, Gilead, Incyte Corporation, Novartis, Roche, and Sobi. Alexander Pohlmann received honoraria from AbbVie, AstraZeneca, BeOne, Eli Lilly, GSK, Sanofi, and Takeda Pharma; received consultancy fees and/or participated in advisory boards for AOP Health, BeOne, Bristol Myers Squibb, Oncopeptides, GSK, and Pfizer, and Takeda Pharma.

## Data Availability

The data that support the findings of this study are available from the corresponding author upon reasonable request. Data sharing is subject to institutional and ethical restrictions due to patient privacy considerations.
